# Preferences of patients with asthma or COPD for treatments in pulmonary rehabilitation

**DOI:** 10.1186/s13561-021-00308-0

**Published:** 2021-04-17

**Authors:** Kathrin Damm, Heidrun Lingner, Katharina Schmidt, Ines Aumann-Suslin, Heike Buhr-Schinner, Jochen van der Meyden, Konrad Schultz

**Affiliations:** 1 Center for Health Economics Research Hannover (CHERH), Leibniz University of Hanover, Member of the German Center for Lung Research (DZL), Otto-Brenner-Str. 7, 30159 Hannover, Germany; 2 Biomedical Research in Endstage and Obstructive Lung Disease Hannover (BREATH), Member of the German Center for Lung Research (DZL), Hannover, Germany; 3 Centre for Public Health and Healthcare, Hannover Medical School, Hannover, Germany; 4 Department of Internal Medicine / Pneumology, Ostseeklinik Schönberg-Holm, Ostseebad Schönberg, Germany; 5 Department of Internal Medicine / Pneumology, Klinik Wehrawald der Bundesversicherungsanstalt für Angestellte Todtmoos, Berlin, Germany; 6 Center for Rehabilitation, Pneumology and Orthopaedics, Klinik Bad Reichenhall, Bad Reichenhall, Germany

**Keywords:** Patient preferences, Asthma, COPD, Chronic obstructive pulmonary disease, Choice-based conjoint analysis, Pulmonary rehabilitation, Latent class model, Mixed-effects model

## Abstract

**Introduction:**

Pulmonary rehabilitation (PR) aims to improve disease control in patients with chronic obstructive pulmonary disease (COPD) and asthma. However, the success of PR-programs depends on the patients’ participation and willingness to cooperate. Taking the patients’ preferences into consideration might improve both of these factors. Accordingly, our study aims to analyze patients’ preferences regarding current rehabilitation approaches in order to deduce and discuss possibilities to further optimize pulmonary rehabilitation.

**Methods and analysis:**

At the end of a 3 weeks in-house PR, patients’ preferences concerning the proposed therapies were assessed during two different time slots (summer 2015 and winter 2015/2016) in three clinics using a choice-based conjoint analysis (CA). Relevant therapy attributes and their levels were identified through literature search and expert interviews. Inclusion criteria were as follows: PR-inpatient with asthma and/or COPD, confirmed diagnosis, age over 18 years, capability to write and read German, written informed consent obtained. The CA analyses comprised a generalized linear mixed-effects model and a latent class mixed logit model.

**Results:**

A total of 542 persons participated in the survey. The most important attribute was sport and exercise therapy. Rehabilitation preferences hardly differed between asthma and COPD patients. Health-related quality of life (HRQoL) as well as time since diagnosis were found to have a significant influence on patients’ rehabilitation preferences.

**Conclusions:**

Patients in pulmonary rehabilitation have preferences regarding specific program components. To increase the adherence to, and thus, the effectiveness of rehabilitation programs, these results must be considered when developing or optimizing PR-programs.

**Supplementary Information:**

The online version contains supplementary material available at 10.1186/s13561-021-00308-0.

## Background

 Thirty million children and adults of 45 years or younger suffer from asthma in Europe, 23 million people suffer from chronic obstructive pulmonary disease (COPD) symptoms [1]. In 2010, COPD was the third leading cause of death worldwide. Despite regularly updated guidelines and available treatments, asthma control continues to be limited [2].

Recurring exacerbations of asthma and COPD are leading to different types of health care consumption, ranging from primary care visits to inpatient or intensive care [3, 4], thus generating high direct and indirect costs up to a total of €34.3 bn for asthma and €48.4 bn for COPD [1]. Many countries strive to reduce the burden of disease and the numbers of exacerbations for the benefit of both, the individual patient and the health care system. To further facilitate comprehensive and effective asthma and COPD management [5, 6], there are promising approaches such as enhancing patient education, and behavior change, empowering patients by ameliorating their understanding of the disease and their physical and psychological condition and enabling them to cope better with long-term care [7]. Pulmonary rehabilitation (PR) is most important for patients with persistent symptoms or limited activity in daily life in spite of adequate outpatient care [8], as it promotes long-term adherence to health-enhancing behaviors and strengthens patients’ empowerment [9]. PR also stops or slows down the disease progression, optimizing functional status and ameliorates patients’ health related quality of life [10–14], while decreasing health care costs in the long run.

However, further research is needed in order to optimize PR [1]. According to Gibson et al. [1], it should be tailored to the needs of patients, for example, by individualizing the intensity and duration of the rehabilitation components.

The success of medical treatments in general, which includes the effect of rehabilitation programs, depends largely on the patients’ motivation, participation, and willingness to cooperate [8]. These aspects could be enhanced by taking the patient’s preferences into account. However, the relative importance of specific rehabilitation components according to pulmonary in-patients remains unknown. Moreover no information is available on the patient-favored structure for a day spent in PR-treatment.

Assessing and analyzing these preferences will allow to rethink present programs and integrate the patients’ perspectives better than before. Therefore, the present study assesses and analyzes the preferences of patients with asthma or COPD regarding different components of pulmonary rehabilitation, including possible social and disease-specific influencing factors of the German context. To enhance the reliability and generate data close to real life, the survey was rolled out in an inpatient setting, as this is the common method by which PR is mostly carried out in Germany.

## Methods

### Choice-based Conjoint Analysis (CA)

We used pairwise comparisons of hypothetical alternatives (i.e. varying rehabilitation options) described by discriminative characteristics (attributes) to measure the influence of these characteristics on patient preferences in a choice-based manner corresponding to the theoretical work of Lancaster [15]. Participants were repeatedly asked to choose between two alternatives [16]. The attributes of these alternatives were classified into different levels, compelling participants to weigh up the pros and cons of each option.

### Identification of attributes

In order to generate clinically meaningful results, identifying relevant attributes is crucial. Input was provided by an earlier quantitative survey that asked 560 patients with asthma and/or COPD at the end of their three-week inpatient program in a rehabilitation center to evaluate the importance of single components of their PR program on a Likert scale ranging between 0 and 10 [17]. Additionally, a literature review was performed using the databases PubMed and Medline (see Supplementary Information), complemented by an “open” internet search that identified, amongst others, guideline-like recommendations for rehabilitation centers. The information gained from the literature research and the quantitative survey was merged and discussed with field experts, such as physicians and clinic managers of rehabilitation centers.

Based on the results, the following final four attributes for the CA were included in the survey: patient education, physical training, respiratory physiotherapy and psychological support. The first three attributes based on the experts’ recommendations. Due to the close link between psychological distress and asthma/COPD in the literature [18, 19], psychological counselling was added as the fourth therapy attribute.

In order to increase the precision of the intended analysis, a realistic and wide range of levels was defined [20–22]. Cognitive overload of the patients with asthma or COPD was avoided restricting the variety to four levels for each attribute and structuring these by time [23]. Presuming that patients could easily picture one treatment day and imagine its structure by hours, we used a time-related attribute level ranging from 0 to 3 h per day.

Each attribute was split into four “delivered time-levels” and the rest of daytime was defined as free time. Optical support was offered as indicated in the graphics in Table 1 in order to simplify the process of choosing between the attributes and the attribute levels for the patients.

**Table 1:**
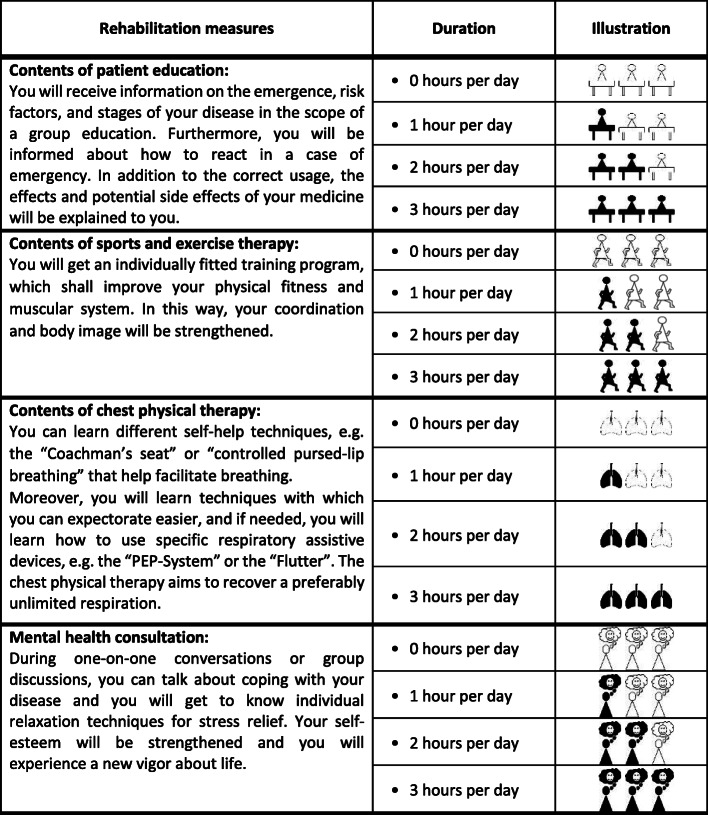
Definition and Illustration of the attributes and the attribute-levels

### Development of the questionnaire

#### Compilation of choice sets

Four attributes with their respective four levels were the starting point for the proposed choice sets (see Table 1). Since not all possible combinations (4^4^ = 256) of treatment profiles could be included, we used the SAS 9.3 software function “%choicEff” maximum-likelihood estimation to set up a d-efficient design for the questionnaire and thus minimized the number of choice sets needed (for further information, see Kuhfeld [24]). Based on the estimated number of choice sets, the sets were split up into two blocks in order to avoid overstraining the participants. The resulting two distinct versions of the questionnaires contained eight choice sets each with two alternatives for every set. Using a paper-pencil questionnaire, participants were asked to choose the rehabilitation program they preferred (A or B).

#### Further information and socio-demographic characteristics

Socio-demographic characteristics, including age, gender, marital status, smoking cessation status before rehabilitation, and employment status, were assessed in order to characterize and describe the study population in depth and to open up options to perform subgroup analyses if required. Information about the duration of their illness, the number of PR components they attended, outpatient trainings or participation in disease management programs were also documented. Additionally, we assessed whether the patients had been allocated to the clinic of their choice and whether there was any participation in rehabilitative treatments not mentioned in the CA. The overall satisfaction with their individual PR was recorded by the last question and their health-related quality of life (HRQoL) was documented via the standardized EQ-5D-5L questionnaire [25].

#### Recruitment of patients and sample size

In each of the contributing centers, all successive patients participating in PR because of their COPD or asthma were invited to answer the questionnaire when fulfilling the inclusion criteria, and written informed consent was obtained. Following a pre-test that checked the questionnaire in terms of comprehensibility and correctness using the think aloud method, the first recruitment and CA-survey was carried out between July and October 2015. The second was carried out with differing consecutive participants between December 2015 and April 2016. Inclusion criteria were asthma and/or COPD being the reason for admission, a confirmed diagnosis upon admission by clinics’ own doctors, at least 18 years old and the ability to write and read German. Patients were recruited to the multicenter study by their respective PR-physicians after completion of at least 2/3 of their rehabilitation program. The questionnaires were answered by the patients on their own, were not personalized, and were collected anonymously in the participating rehabilitation clinics using a post-box in a public space.

The sample was recruited from three rehabilitation centers in Germany located in different landscapes: mountains, seaside, and near brine springs. All three clinics are accredited and specialized in in-house rehabilitations for patients with asthma or COPD. Following Johnson and Orme’s rule of thumb [26, 27], we estimated the total of the required sample size to be *n* = 500.

#### Data analysis

Digitalization and data entry were performed by two independent persons in the study center. All CA data were first effect coded (preparation step). The choice of therapy alternative (binary coded) was set as the dependent variable; independent variables were rehabilitation attributes and socio-demographic variables, disease-specific variables, and HRQoL. Descriptive analyses were used to confirm the data structure, identify missing data and assess the distribution of the different attributes’ levels and persons’ individual characteristics. A logit regression model was chosen corresponding to the data structure and correlations between the attributes [28]. The best model was identified by goodness-of-fit. All analyses were performed using the R statistics software and the “survival” package by Therneau (2015) [29]. Difference between asthma and COPD patients’ preferences were considered in subgroup analyses and possible gender differences were addressed.

The descriptive analyses performed on a cleaned dataset included the mean, median, with their standard deviations (SD) and the percentages of sample characteristics for the distribution of participants. Furthermore, we analyzed correlations by Pearson product-moment correlation coefficient (linear relationship between normally distributed variables) or Spearman rank correlation coefficient (rank ordered variables) between the variables in a correlation plot. Correlations of ± ≥0.5 moderate to strong associations between the tested variables [30]. Consequently, highly correlated variables should not be used together in further multivariate regression models, unless they are integrated as interaction effects. Furthermore, we analyzed the Likert scale ratings with regard to the importance of attributes.

The CA tasks were analyzed with logistic regression models, and the associations between the level attributes (independent variables) and the choice of profile (dependent variable) were evaluated. The used formulae are displayed below.

*Formula 1: Utility function and choice model.*

Step 1) Utility function

1 $$ {U}_i=V\left(\beta, {X}_i\right)+{\varepsilon}_i $$

Step 2) Choice of profile (logit function)

2 $$ \mathit{\Pr}\left( choice=i\right)=\frac{e^{V\left(\beta, {X}_i\right)}}{\sum_j{e}^{V\left(\beta, j\right)}} $$

**Table:** 

Explanations:	
*U*: utility of alternative i	
*V*(*β*, *X*_*i*_): explainable component of utility, defined by the attribute levels X_i_ and β	
*εi*: non-explainable or random component of utility	
*X*_*i*_: vector of attribute levels for alternative i	
*i*: one alternative from j	
j: set of alternatives including i	
*β*: vector of estimated coefficients (preference weight)	

Source: based on Hauber et al. [31]

The first type of model was a generalized linear mixed-effects model (GLMM). It included the effects of paired choices for each person due to the random effects of number of choice sets and the person identification number (serial, PERSID). The choice model for the GLMM is displayed in the following (Formula 2).

*Formula 2: Generalized linear mixed-effects model*

3 $$ \mathit{\Pr}\left( choice=i\right)=\frac{e^{V\left({\tilde{\beta}}_n,{X}_i\right)}}{\sum_j{e}^{V\left({\tilde{\beta}}_n,j\right)}} $$

with.

4 $$ {\displaystyle \begin{array}{l}{\tilde{\beta}}_n=f\left(\beta, \sigma \left|{V}_n\right.\right)={\upbeta}_1\ast \mathrm{SCHOOL}\kern0.5em +\kern0.5em {\upbeta}_2\ast \mathrm{SPORTS}\kern0.5em +\kern0.5em {\upbeta}_3\ast \mathrm{CHEST}\kern0.5em +\kern0.5em {\upbeta}_4\ast \mathrm{MENTAL}\kern0.5em +\\ {}\upsigma \ast \left(\mathrm{serial}+\mathrm{PERSID}\right)\kern0.5em +\kern0.5em {\upbeta}_0\end{array}} $$

**Table:** 

Explanations:	
$\overset{\sim }{\beta_n}$ choice set and person specific estimated preference weights	
*σ*: standard deviation of preferences due to individual characteristics of the sample	
n: choice set and person specific component	

Source: based on Hauber et al. [31]

The second type of model was a latent class mixed logit model (LCMLM). This model type is based on preference estimates for different groups of participants (latent classes) (Eqs. 5, 6 and 7). The latent class-membership is based strictly on the collected data. There are coefficients for the preference weights of the classes and the class-membership effects. The following variables were tested as class-membership effects: age, gender, disease type, clinic, survey wave, experiences with rehabilitation or patient education programs, disease duration, marital status, employment status, HRQoL, and satisfaction with rehabilitation program. The final model is presented below (Eq. 7)

*Formula 3: Latent class mixed logit model*

5 $$ \mathit{\Pr}\left( choice= 1\right)={\sum}_q\kern0.5em \mathit{\Pr}\left( choice=i\left|{\tilde{\beta}}_q\right.\right){\pi}_q $$

with

6 $$ \mathit{\Pr}\left( choice=i\left|{\tilde{\beta}}_q\right.\right)=\frac{e^{V\left({\tilde{\beta}}_q,{X}_i\right)}}{\sum_j{e}^{V\left({\tilde{\beta}}_q,{X}_j\right)}} $$

with.

7 $$ {\displaystyle \begin{array}{l}{\tilde{\beta}}_q=f\left(\beta, \sigma \left|{V}_q\right.\right)={\upbeta}_1\ast \mathrm{SCHOOL}\kern0.5em +\kern0.5em {\upbeta}_2\ast \mathrm{SPORTS}\kern0.5em +\kern0.5em {\upbeta}_3\ast \mathrm{CHEST}\kern0.5em +\kern0.5em {\upbeta}_4\ast \mathrm{MENTAL}\kern0.5em +\\ {}\upsigma \ast \left(\mathrm{serial}\kern0.5em +\kern0.5em \mathrm{age}\kern0.5em +\kern0.5em \mathrm{gender}\kern0.5em +\kern0.5em \mathrm{diagn}\kern0.5em +\kern0.5em \mathrm{dur}\kern0.5em +\kern0.5em \mathrm{EQ}5\mathrm{DIndex}\right)\kern0.5em +\kern0.5em {\upbeta}_0\end{array}} $$

**Table:** 

Explanations:	
q: class specific component	
*π*_*q*_: probability of being in one of the different classes	

Source: based on Hauber et al. [31]

The β-coefficients resulting from GLMM and LCMLM showed the preference weights for the attribute levels. Preference weights greater than 0 indicate positive preferences and weights smaller than 0 indicate negative preferences or disfavor of the attributes. All coefficients were assumed to be significant at α ≤ 0.05. Different models for each model type were tested. The model with the best fit due to Akaike (AIC) and Bayesian information criteria (BIC) was chosen. All analyses were performed with R statistics 3.1.2, “lme4” (for GLMM), and “lcmm” (for LCMLM) packages.

## Results

### Descriptive results

Among the total number of 542 participants, (see Table 2), 54.37% of the patients suffered from asthma and 35.90% from COPD. The median age of the total sample was 55 years. The asthma rehabilitants were younger than the COPD patients (53 years vs. 58 years). In the subsample of participants with COPD, the proportion of women was higher than in the sample of patients with asthma (COPD: 54.89% female vs. Asthma: 44.85% female), whereas in the total sample, the relationship between male and female patients was almost balanced (total: 48.86% female). The COPD subsample had a higher proportion of smokers and former smokers compared to the one of patients with asthma. The satisfaction with the rehabilitation program was higher in patients with asthma than in the COPD cohort (5 vs. 4). The HRQoL (EQ-5D-5L Index) was higher in patients with asthma than in patients with COPD (0.89 vs. 0.82) (Table 2).

**Table 2:** Sample characteristics

Characteristics	Total	Asthma	COPD
Sample size	542	279	187
Asthma or COPD	51.7% asthma	100% asthma	100% COPD
	34.7% COPD		
	9.4% both		
Median age (SD)	55 (8.96)	53 (9.46)	58 (7.03)
Gender	48.86% female	44.85% female	54.89% female
Smoking status	50.04% non-smoker	72.21% non-smoker	25.06% non-smoker
	24.14% smoker	11.47% smoker	41.44% smoker
	25.82% former smoker	16.32% former smoker	33.50% former smoker
Participation in DMP	51.62% no	52.24% no	53.02% no
	39.39% yes	41.19% yes	35.56% yes
	8.99% don’t know	5.85% don’t know	11.42% don’t know
Median satisfaction with rehabilitation program (SD) *scale 1–5*	4 (0.84)	5 (0.83)	4 (0.84)
Median HRQoL (EQ-5D-5L Index) (SD) *scale 0–1*	0.89 (0.14)	0.89 (0.13)	0.82 (0.15)

**Fig. 1:**
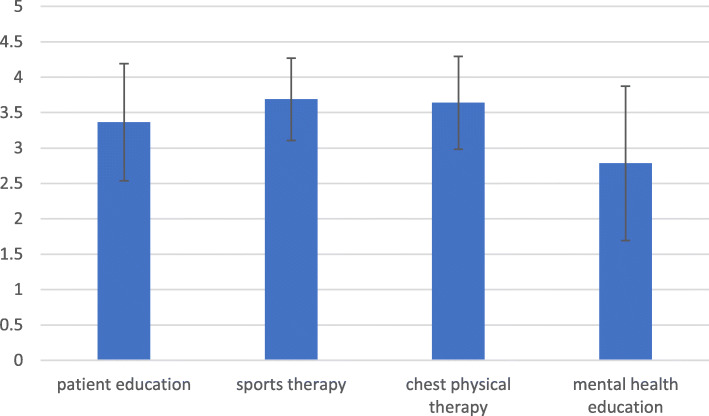
Evaluation of rehabilitation components (Likert Scale). Blue bars show the mean value, and black error indicators show the standard deviation

The results (means and SD) of the attribute rating on a Likert scale are presented in Fig. 1. According to the ratings, sports therapy (3.69, SD: 0.58), followed by chest physical therapy (3.64, SD: 0.65), was perceived as the most important rehabilitation component. The third ranked patient education was assigned a value of 3.37 (SD: 0.83). Mental health consultation with a value of 2.78 (SD: 1.09) was rated the last important attribute of the fictional PR. This attribute had the broadest SD.

As potential covariates for correlation effects, the following variables were tested: gender, age, marital status, employment status, smoking status, hobbies, diagnosis (asthma or COPD), time of diagnosis, desired location indicated, admission to the desired location, satisfaction with the recently undergone PR, difficulties with answering the questionnaire, EQ 5D-Index, and experiences with patient education overall. Only one correlation was identified: the one between age and employment status.

### Multivariate models

#### Mixed logit model

**Fig. 2:**
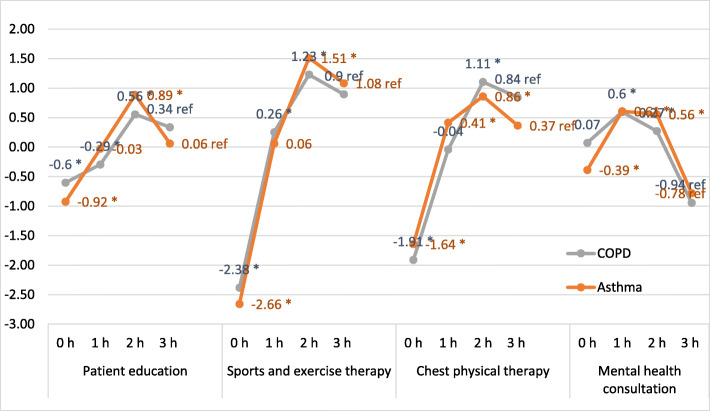
Preferences of rehabilitants (mixed logit model). Note. Mixed-effects: PERSID and serial. h stands for hours

The preferences of the patients with asthma or COPD regarding the rehabilitation components estimated by the mixed logit model are shown in Fig. 2. There were only minor differences between the two groups of asthma and COPD rehabilitants. In both subgroups, the most preferred attribute level was 2 h of sports therapy (β_asthma_ = 1.51, *p* < 0.01; β_COPD_ = 1.23, *p* < 0.01). Deviating preferences were found for patient education: whereas asthma patients expressed stronger dislike for zero hours of patient education stronger than did COPD patients (β_asthma_ = − 0.92; β_COPD_ = − 0.60, both *p* < 0.001), the COPD patients expressed stronger preference for 3 h of patient education than did asthma patients (β_asthma_ = 0.06; β_COPD_ = 0.33). Furthermore, both groups of rehabilitants preferred 2 h of chest physical therapy . However, there was a slight difference in the attributed value: patients with asthma preferred 2 h less strongly than did COPD patients (β_asthma_ = 0.86, *p* < 0.01; β_COPD_ = 1.11, *p* < 0.01). Both groups were fond of 1 h of mental health consultation (β_asthma_ = 0.61; β_COPD_ = 0.6, both *p* < 0.001) (Table 3).

**Table 3:** Mixed logit model results

	COPD			Asthma		
	Estimate	SE	*p*-value	Estimate	SE	*p*-value
Patient education						
0 h	− 0.6	0.109	0	−0.92	0.0945	0
1 h	−0.29	0.1286	0.0226	−0.03	0.1005	0.798
2 h	0.56	0.0988	0	0.89	0.0823	0
3 h	0.33	ref	ref	0.06	ref	ref
Sports and exercise therapy						
0 h	−2.38	0.1289	0	−2.66	0.1113	0
1 h	0.26	0.0959	0.0078	0.06	0.0785	0.428
2 h	1.23	0.1068	0	1.51	0.0988	0
3 h	0.89	ref	ref	1.09	ref	ref
Chest physical therapy						
0 h	−1.91	0.1122	0	−1.64	0.1008	0
1 h	−0.04	0.1091	0.7189	0.41	0.0878	0
2 h	1.11	0.112	0	0.86	0.0889	0
3 h	0.84	ref	ref	0.37	ref	ref
Mental health consultation						
0 h	0.07	0.1238	0.5499	−0.39	0.096	0.0001
1 h	0.6	0.1082	0	0.61	0.08	0
2 h	0.27	0.1005	0.0067	0.56	0.0901	0
3 h	−0.94	ref	ref	− 0.78	ref	ref
(Intercept)	0.0557	0.8693	0.3387	0.0096	0.9773	0.3357

Note: h stands for hours

#### Latent class mixed logit model

**Fig. 3:**
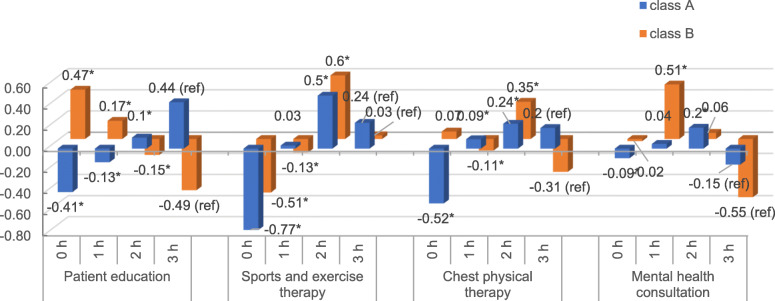
Results from the latent class mixed logit model. Note: h stands for hours

The data set showed two latent classes: The first class (A, blue bars in Fig. 3) preferred 2 h of sports therapy (β_cl1,SPO2_ = 0.5, *p* < 0.001) and disfavored zero hours of sports therapy the most (β_cl1,SPO0_ = − 0.77, *p* < 0.001). In addition, they preferred 3 h of patient education, 2 h of chest physical therapy, and 2 h of mental health consultation. Class B also preferred 2 h of sports therapy the most (β_cl2,SPO2_ = 0.6, *p* < 0.001), and they also showed high positive interest for 2 h of chest physical therapy (β_cl2,CPT2_ = 0.35, *p* < 0.001). However, in contrast to Class A, Class B weighed zero hours of patient education higher than 3 h. Overall, the preferences for sports therapy are almost the same in all three levels compared to Class A (β_cl2,SPO0_ = − 0.51, β_cl2,SPO1_ = − 0.13, β_cl2,SPO2_ = 0.6, β_cl2,SPO3_ = 0.03). Additionally, Class B preferred 1 h of mental health consultation (β_cl2,MH1_ = 0.51), which is also different from Class A.

The class-membership effects of the LCMM showed that a higher proportion of rehabilitants was assigned to Class A than to Class B (n_cl1_ = 357 [83.22%] and n_cl2_ = 72 [16.78%]). Class A differed significantly from Class B regarding HRQoL and time since diagnosis (Table 4): it comprised a higher proportion of rehabilitants with worse HRQoL and less time since diagnosis. Age, gender, and type of disease (asthma or COPD) showed no significant differences between the classes.

**Table 4:** Class-membership effects in the latent class mixed logit model

	Class A		
Fixed effects class-membership model	Coefficient	Standard error	***p***-value
Intercept	**6.074**	**1.578**	**0.000**
Age (mean centered)	0.014	0.017	0.399
Gender (ref = male)	−0.469	0.291	0.107
COPD (ref = Asthma)	−0.244	0.357	0.495
Time since diagnosis	**−0.027**	**0.011**	**0.012**
EQ-5D Index	**−3.705**	**1.431**	**0.010**

## Discussion

 Scientific research is increasingly focused on the treatment preferences of patients with lung diseases [32]. Several surveys used conjoint analyses or choice experiments thus demonstrating the acceptance and usability of tradeoff methods in this field: while some studies have assessed the importance of different disease management features such as treatment simplicity, the number of medications, or the dosage of steroids [33, 34], others documented preferences for specific products (e.g. inhalers) [35, 36], the location of care [37], or the willingness-to-pay for care at different symptom levels [38, 39].

However, no studies so far have investigated the preferences of patients with asthma or COPD regarding the components of their PR. Moreover, to our knowledge, none of these studies have taken into consideration possible social and disease-specific influencing factors. A CA was used to determine these preferences.

Summarizing, the survey data showed the following results:

1. Rehabilitation preferences differed hardly between patients with asthma and COPD.
2. The most important attribute influencing the PR program choice was sport and exercise therapy. While "zero hours" of sports per day had the strongest negative influence on the participants’ choices, 2 h of sports per day had the most positive impact on the patients choice.
3. HRQoL as well as the duration of illness had a significant influence on the patient’s rehabilitation preferences, especially regarding non-physical components.
4. Independent of the thematic succession, the most preferred daily PR-treatment was a combination of 2 h of patient education, 2 h of sports therapy, 2 h of chest physical therapy and 1 h of mental health consultation.

While Wijnen et al. [40] reported a difference in the resulting preferences when measured with rating scale or trade-off decisions, the findings of our survey identified no differences between Likert scale and DCE results. Both procedures showed mental health consultation to be considered least important compared to all the other attributes. This finding supports previous results of surveys using only a Likert scale as assessment tool [17]. While all PR-components were rated as at least “rather important,” psychological counselling was among the three lowest rated PR-components. Education, sports and chest physical therapy were at the top of the rating list. The "cancer-patients" in the survey of Faller et al. [41] also attributed less relevance to psychological issues when assessing their expectations concerning their rehabilitation goals. 

Bethge and Wienert et al. [42, 43] conducted two discrete choice experiments (DCE) regarding rehabilitation components in the fields of orthopedics and oncology. However, their surveys focused work-related aspects such as stress management and workplace training, whilst our experiment assessed preferences concerning pneumological rehabilitation components. Geidl et al. [44] performed a DCE measuring stroke patients’ exercise preferences and demonstrated the adversity of the patients for the two extremes: none or high physical activity. Participants strongly favored light and moderate to intense physical activity. Our data suggest similar tendencies. However, subgroups analysis in our experiment revealed a difference between the “Class A” and “Class B”. Patients in Class A, who had a higher probability of a worse HRQoL and were diagnosed positive since a shorter period of time displayed much higher preferences for the educational components of the PR program than “Class B”. In particular, they preferred the maximum number of hours (=3 h) of patient education and 2 h of mental health consultation. In contrast, Class B fancied no patient education at all (zero hours) and only 1 h of mental health consultation. Moreover, they strongly disfavored 3 h of both components. Patients belonging to Class A felt a greater need to understand the disease and learn how to deal with it than did patients of Class B. In the above-mentioned survey by Linger et al. [17], HRQoL was found to have a significant impact on the ratings. Patients with a lower HRQoL at the beginning of their rehabilitation considered education and mental counseling as more important than patients with better HRQoL did.

Additionally, Class B demonstrated a very clear preference for 2 h of sports and 2 h of chest physical therapy. The results are even more distinct in Class A: For these patients, 3 h (max) of both components had a relevant positive impact on their choice. Hence, in addition to the educational contents of PR, they also showed a tendency towards more hours of physical training (sports and chest) compared to Class B. The “type of disease” was not a factor differentiating between the classes or subgroups thus making it difficult to formulate distinct preference-based recommendations for patients with asthma or with COPD.

Respecting both equally: the medical necessities and the patients’ preferences appears to be a promising approach when aiming to increase the effectiveness of rehabilitation programs [44]. Effectiveness is known to largely depend on the patients’ motivation and willingness to cooperate in PR programs. Our survey focused on inpatient PR. All of the survey participants had experienced the PR-programs in question beforehand and were used to freely dispose of their time budget. The choice tasks during the survey were tradeoff decisions between hours with learning contents or sports units and leisure time. The following result strongly advocates against the possible PR-focus of maximizing leisure time: participants preferred a daily combination of 2 h of patient education, 2 h of sports therapy, 2 h of chest physical therapy, and 1 h of mental health consultation to the minimum amount of all these program parts.The negative coefficients of the attribute levels “zero hours” underpins the patients’ motivation to use the PR in a very active manner even further.

The differences between the subgroups in our survey demonstrated the necessity to discriminate patient preferences carefully: they are not homogeneous or average values but might be highly specific in (latent) subgroups as well. Correspondingly Soekhai et al. [45] recently documented an increase in DCE using subgroup-specific or subgroup-identifying analysis methods such as latent class regressions.

Our results could be used to enhance the adherence to PR in patients with asthma or COPD. Existing PR-programms should be adjusted in accordance with our findings by incorporating the pattern of preference-based components discussed above while considering the patients’ views of the appropriate “time per rehab measure.” This may lead to further comprehensive improvement of the positive overall consequences of PR for the two most common respiratory diseases. In the future, patient preferences should be assessed before starting a PR program. Moreover, changes in the patients’ perspective over time during PR should be documented closely in order to better understand the impact of the patients’ expectation, estimation and appraisal on PR success and possibly adapt treatment guidelines accordingly.

### Limitations

This study is the first one to use a choice-based conjoint analysis to assess and analyze preferences of patients with asthma or COPD for treatment components in PR. By carefully analyzing and interpreting our findings, we tried to identify common rules that could be generalized and extended to other indications.

Patients were invited to participate in the study by their physicians after completion of at least the first 2/3 of their inpatient rehabilitation program. Although this procedure resulted in a high response rate it could possibly have caused a response bias: Patients might fear that their responses could be perceived as negative by their treating physicians and thus affect the future medical treatment negatively. To reduce this bias patients were informed in advance about the intended use of the data, the data collection setting and the de facto anonymity of the questionnaire. A post-box in a public space was used to collect the printed forms.

In order to outrun a “seasonal influence” bias, data were collected during a summertime slot and a winter time-one. Moreover, data collection was performed in several study centers all over Germany in topographically different areas to overcome a geographical bias too. Both factors are known to influence patients’ perceived well-being and the severity of their disease-related symptoms. The German health care system could be considered as a further source of limitation for the representability of the sample and the subsequent survey results. The data collection was performed in a German setting where PR is usually carried out on an in-patient basis thus restricting the international applicability of the findings, where outpatient PR programs are more common. However, the different choice sets of our survey offer hypothetical scenarios which could be used in other countries whenever the therapy components of rehabilitation are similar to the attributes included in the CA.

As our survey only assessed the patients view, it lacks information about possibly relevant other medical influencing factors, such as the severity of symptoms or comorbidities (e.g. depression). These factors may be important for a deeper understanding of subgroup effects,- thus future research should include additional medical information provided by physicians and other center-based health care workers.

It would have been advantageous toconsider cost-related attributes (e.g. additional payment) [46] when interpreting our results. However, in the German healthcare system additional payments (in relevant amounts) are rather unusual and therefore difficult to estimate. Moreover, asking patients directly about their willingness to pay for care and the acceptable rate of fees would have possibly unsettled the participants. This had to be imperatively avoided with respect to the recruiting study centers.

The German rehabilitation components focus on recovery and prevention, especially through educational contents. The (long-term) effectiveness of the PR depends largely on the effort the patient is willing to contribute. Thus, participants of our survey were questioned about their preferred PR daily routine, with a tradeoff between treatments components and leisure time.

## Conclusions

The number of hours of sports and exercise therapy had the greatest impact on the patients’ preference for or against a rehabilitation program. Preferences regarding non-physical components such as education and mental health consultation differed between subgroups. To increase the adherence to and thus the effectiveness of rehabilitation programs, these results must be considered when developing or optimizing PR programs and treatment guidelines.

## Supplementary Information

**Additional file 1.**

## Data Availability

The data that support the findings are not publicly available, as the publication of the collected primary data is not covered by the informed consent.

## Abbreviations

CA: Choice-based Conjoint Analysis
COPD: Chronic Obstructive Pulmonary Disease
DCE: Discrete Choice Experiment
DMP: Disease Management Program
PR: Pulmonary Rehabilitation
HRQoL: Health-Related Quality of Life
