# Supplementary material for: Preferences of patients with asthma or COPD for treatments in pulmonary rehabilitation
Source: Health Econ Rev. 2021 Apr 17;11:14. doi: 10.1186/s13561-021-00308-0 (PMC8053281; doi:10.1186/s13561-021-00308-0)
Supplement: Supplementary file 1 — Additional file 1. [file 13561_2021_308_MOESM1_ESM.docx]

**Supplementary Information**

A systematic literature and guidelines review was performed in the database PubMed and Medline to identify (further) attributes in January 2015. It used the following English search terms combined with AND: (i) (preference OR preferences OR priorities OR priority); (ii) (rehabilitation); (iii) (asthma or COPD or chronic obstructive pulmonary disease). The search identified 125 records, which were screened by titles and abstracts. Thus, 117 publications were excluded and 8 full text articles were assessed for eligibility. All studies that analysed the preferences of patients with asthma or COPD during rehabilitation were included. Nevertheless, none of the eight full text articles fulfilled the inclusion criteria.

Since not all studies and relevant information might be published in scientific databases, an additional “open” internet search was performed identifying amongst others guidelines-like recommendations which a rehabilitation centre/clinic has to follow (at least) in order to receive reimbursement for their work from the health insurance companies in Germany. According to these guidelines, each rehabilitation program contains main therapy components like physical training therapy (e.g. endurance training, strength training), respiratory physiotherapy, structured patient education, psychosocial support and regulated adjustment of medication [1]. Additional further components include but are not limited to, nutrition consultation, smoking cessation therapy, ergotherapy and mucolytic physiotherapy.

1. Bundesarbeitsgemeinschaft für Rehabilitation e. V. Rahmenempfehlungen zur ambulanten pneumologischen Rehabilitation vom 06. Juni 2008 2009. Available at: https://www.vdek.com/vertragspartner/vorsorge-rehabilitation/amb_reha/bar-konzept/_jcr_content/par/download_2/file.res/re_amb_pneumo_reha.pdf. Accessed September 02, 2019.
